# Interactions between Spider Silk and Cells – NIH/3T3 Fibroblasts Seeded on Miniature Weaving Frames

**DOI:** 10.1371/journal.pone.0012032

**Published:** 2010-08-09

**Authors:** Joern W. Kuhbier, Christina Allmeling, Kerstin Reimers, Anja Hillmer, Cornelia Kasper, Bjoern Menger, Gudrun Brandes, Merlin Guggenheim, Peter M. Vogt

**Affiliations:** 1 Department of Plastic, Hand and Reconstructive Surgery, Hannover Medical School, Hannover, Germany; 2 Institute of Technical Chemistry, Leibniz University of Hannover, Hannover, Germany; 3 Institute of Cell Biology and Electron Microscopy, Center of Anatomy, Hannover Medical School, Hannover, Germany; 4 Division of Reconstructive Surgery, Department of Surgery, University Hospital Zürich, Zürich, Switzerland; Institut Européen de Chimie et Biologie, France

## Abstract

**Background:**

Several materials have been used for tissue engineering purposes, since the ideal matrix depends on the desired tissue. Silk biomaterials have come to focus due to their great mechanical properties. As untreated silkworm silk has been found to be quite immunogenic, an alternative could be spider silk. Not only does it own unique mechanical properties, its biocompatibility has been shown already in vivo. In our study, we used native spider dragline silk which is known as the strongest fibre in nature.

**Methodology/Principal Findings:**

Steel frames were originally designed and manufactured and woven with spider silk, harvesting dragline silk directly out of the animal. After sterilization, scaffolds were seeded with fibroblasts to analyse cell proliferation and adhesion. Analysis of cell morphology and actin filament alignment clearly revealed adherence. Proliferation was measured by cell count as well as determination of relative fluorescence each after 1, 2, 3, and 5 days. Cell counts for native spider silk were also compared with those for trypsin-digested spider silk. Spider silk specimens displayed less proliferation than collagen- and fibronectin-coated cover slips, enzymatic treatment reduced adhesion and proliferation rates tendentially though not significantly. Nevertheless, proliferation could be proven with high significance (p<0.01).

**Conclusion/Significance:**

Native spider silk does not require any modification to its application as a biomaterial that can rival any artificial material in terms of cell growth promoting properties. We could show adhesion mechanics on intracellular level. Additionally, proliferation kinetics were higher than in enzymatically digested controls, indicating that spider silk does not require modification. Recent findings concerning reduction of cell proliferation after exposure could not be met. As biotechnological production of the hierarchical composition of native spider silk fibres is still a challenge, our study has a pioneer role in researching cellular mechanics on native spider silk fibres.

## Introduction

A plethora of biomaterials used as scaffolds for tissue engineering as well as their influence on the quality of the generated tissue according to their specific properties have been described previously. It has been discussed that, foremost, an ideal tissue-engineering scaffold should act as replacement for the tissue that should be restored and consequently have comparable mechanical attributes [1]. It should bridge the gap, carry histologically typical cells and guide tissue repair. Subsequently, it should be degraded and replaced completely by newly grown tissue without exerting too much influence on the environment, e.g. via pH-changes.

Mainly, research has focused on the use of degradable scaffold materials, especially synthetic polymers like polyglycolic acid (PGA) or polylactic acid (PLA) [2]. While these often promise very good moldability, they often have poor mechanical properties.

For example, PGA scaffold for nerve regeneration showed elongation and partial collapse [3], while the use of PLA scaffolds resulted in rapid degradation in vivo, generating acidic degradation products that altered the pH [2]. This hydrolysation decreased the regeneration process, as evidenced by a lack in the number of sprouting axons. Collagen, by contrast, is decomposed in a neutral milieu, but loses its mechanical properties during the digesting process, if not appropriately stabilized, e.g. by cross-linking of the individual polymer strains [4]. Nevertheless, cross-linking substantially alters the collagens' properties and tissue responses are thus apparently altered compared to the native protein [5].

While silkworm silk from *Bombyx mori* has been used extensively in biomedical applications [6]–[9], spider silk has barely been researched, although it offers impressive mechanical and structural properties. Dragline silk from *Nephila clavipes* provides an excellent combination of light weight (1.3 g/cm^3^), tensile strength (up to 4.8 GPa as the strongest fibre known in nature) and remarkable toughness and elasticity (up to 35%) [10], [11]. Notably, it is also sterilizable because of its high temperature resistance (approximately around 250°C) [12], [13]. Another astonishing property of spider dragline silk is the so-called supercontraction: Putting spider silk fibres in water, a structural contraction resulting in a loss of length of more than 50% can be observed [14], [15]. Studies by Sponner et al. revealed that native spider silk is built out of five layers, which can each be differentiated into an outer shell and an inner core [16].

As the mechanical properties of the silk and the biochemistry of the silk protein have been clarified in the past years, much effort has been invested in the biotechnological production of a comparable silk. Yet there are very few articles dealing with tissue engineering purposes [17], [18]. Summarizing the state of the art, Brini et al. used genetically modified silk [19], concluding that modification of the dissolved spider silk protein with arginine-glutamine-asparagine-(RGD)-fragments enhances cell growth. Gellnyck et al. described cell growth on scaffolds produced by freeze-drying and salt-leaching of an aqueous solution of dissolved egg sac silk, either with or without enzymatic treatment with trypsin or proteinase K [20]. Another field of application was the use of native spider silk fibres for living nerve conduits [21]. Schwann cell seeded nerve conduits have been also used in sciatic nerve regeneration [22].

Biocompatibility was demonstrated in a study in which dragline silk was implanted subcutaneously in pigs. Immunoreactions were comparable to fibrous silk polymers and established wound dressings like polyurethane, collagen or gauze [23]. In the long-term investigations, the fibrotic response was even superior to Vicryl® sutures (Ethicon, Sommerville, NJ, USA), although here egg sac silk or enzymatically treated egg sac silk were used, respectively [24]. Another attempt focussed on a biotechnologically produced spider silk-elastin, which increased the proliferation rate of human chondrocytes if coated to a polystyrene surface [25]. In contrast, a recent study displayed a decrease in proliferation rates of endothelian cells exposed to *Nephila edulis* spider silk [26].

**Figure 1:**
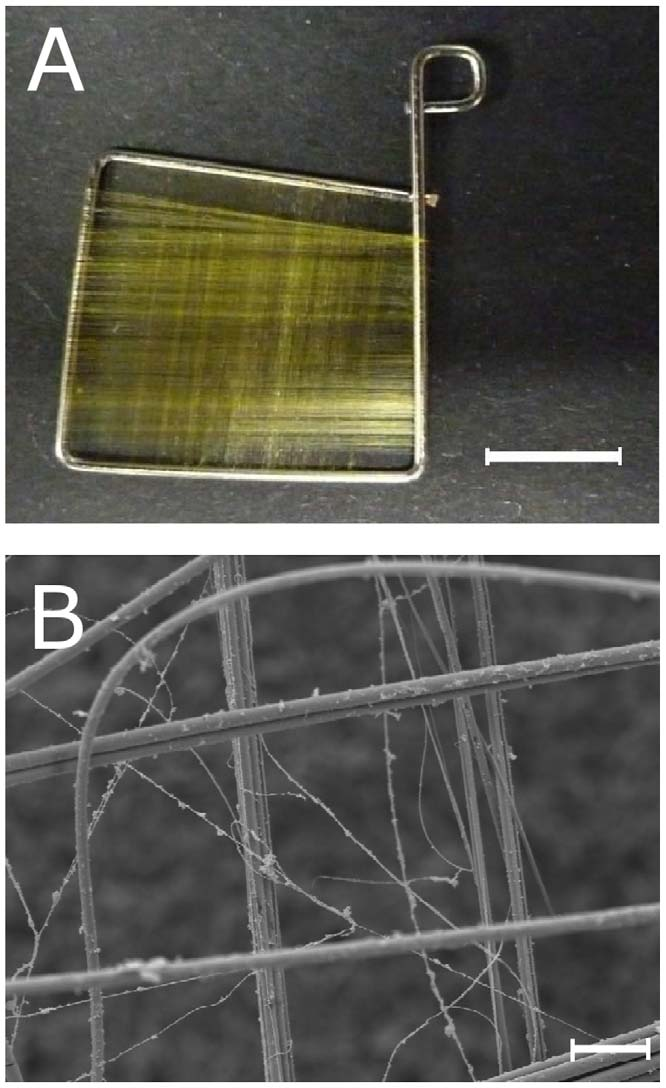
Appearance of the scaffolds used in the study. A: Photography of a weaving frame used in this study, made of stainless dental steel with 0.7 mm diameter, bended by the authors; scale bar represents 5 mm. B: SEM of a weaving frame used in this study, weaved with spider silk; magnitude ×600, scale bar represents 20 µm.

While these findings show an ambivalence concerning the suitability of spider silk for biomedical applications, the purpose of this study was to analyze cell growth on native spider silk by investigation of adhesion, proliferation and migration of NIH/3T3 fibroblasts. As we wanted to avoid changes in the surface properties of the silk fibres due to cross-linking or solubilization, we had to invent a method to design scaffolds for native dragline silk without destroying its fibre structure (Fig. 1). Additionally, the aim was to avoid complex structures like they occur in egg sac cocoons but to provide a certain two-dimensionality without the disadvantage of supercontraction [14], [15], [27]. Proliferation was measured in comparison to trypsin digested silk fibres, which resulted in an alteration of the biocompatibility in the studies by Gellnyck et al. [24].

## Results

### Morphologic analysis

**Figure 2:**
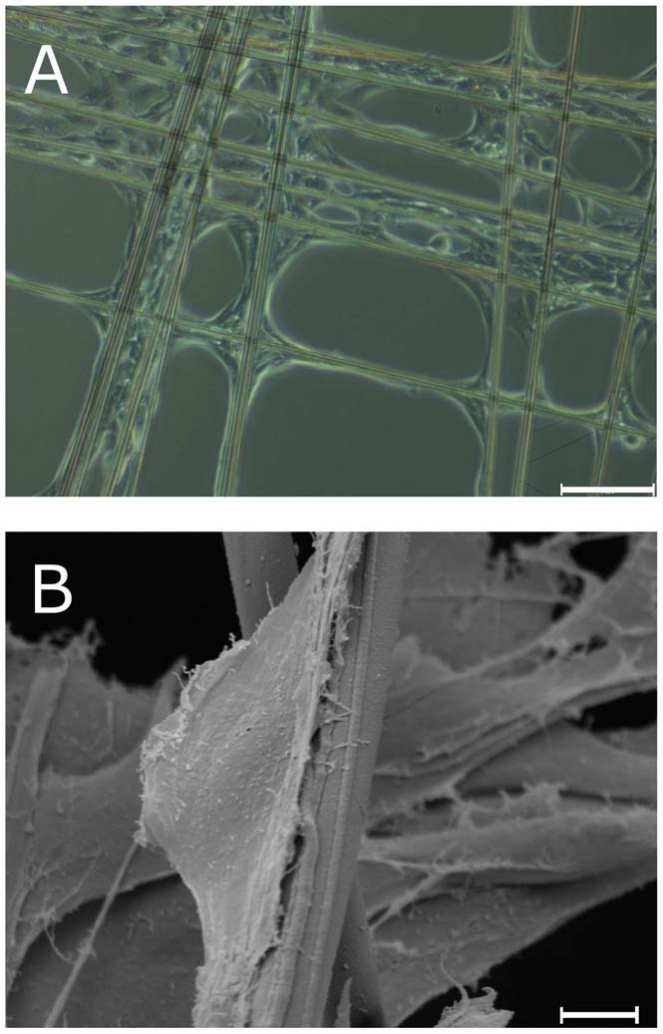
Cell attachment to spider silk fibres on day 1. A: Light microscopy of fibroblasts adhering on spider silk fibres assembled in crosslink pattern; magnitude ×100, scale bar represents 50 µm. B: SEM of a single fibroblast sticking to a fibre, showing broad-base spindle shape; magnitude ×2840, scale bar represents 5 µm.

Light microscopy on day 1 (Fig. 2a) already showed cell adhesion and spreading. Cells were adhesive and spread out with the broad base attached to the silk fibre, indicating adhesion to the fibre [28].

The spindle-shaped and asymmetric morphology of a single fibroblast in the more detailed SEM revealed a polarity with a more and a less convex side of the fibroblast, which is defined as part of cell migration processes (Fig. 2b) [29].

**Figure 3:**
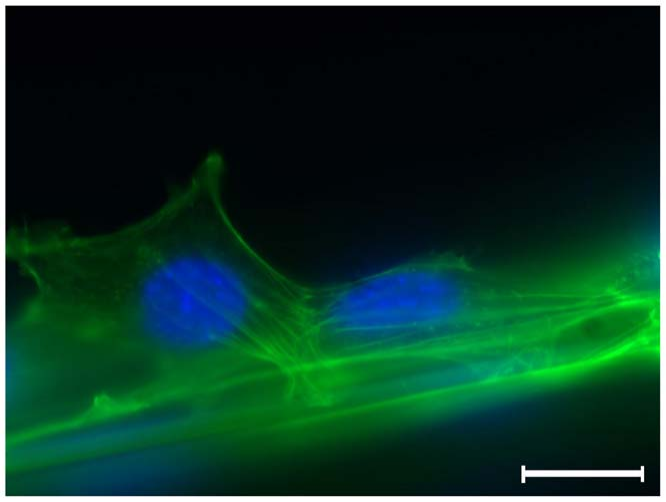
Immunofluorescence microscopy of fibroblasts adhering to a spider silk fibre. Fibroblasts are sticking to fibre, note the orientation of intracellular actin filament bundles, indicating the direction of forces; DAPI staining of cell nuclei in *blue*, α-actin as well as autofluorescence of spider silk in *green*; magnitude ×400, scale bar represents 10 µm.

These findings could be confirmed by analyzing the assembly of the actin filament bundles, which could be regarded as intracellular lines of force (Fig. 3): Concentration of the actin cortex inside the lamellopodium was observed, which counts as characteristic for migratory processes (Fig. 3, cell on the right).

Interestingly, treatment with Pluronic F-127, which inhibts cell-adhesion by blocking hydrophilic binding sites of common cell culture substrates like polystyrene or polyethylene, reduced the number of fibroblasts on the bottom of the cell culture plates while the silk seeding remained unaffected (data not shown).

### Proof of viability and cell attachment

**Figure 4:**
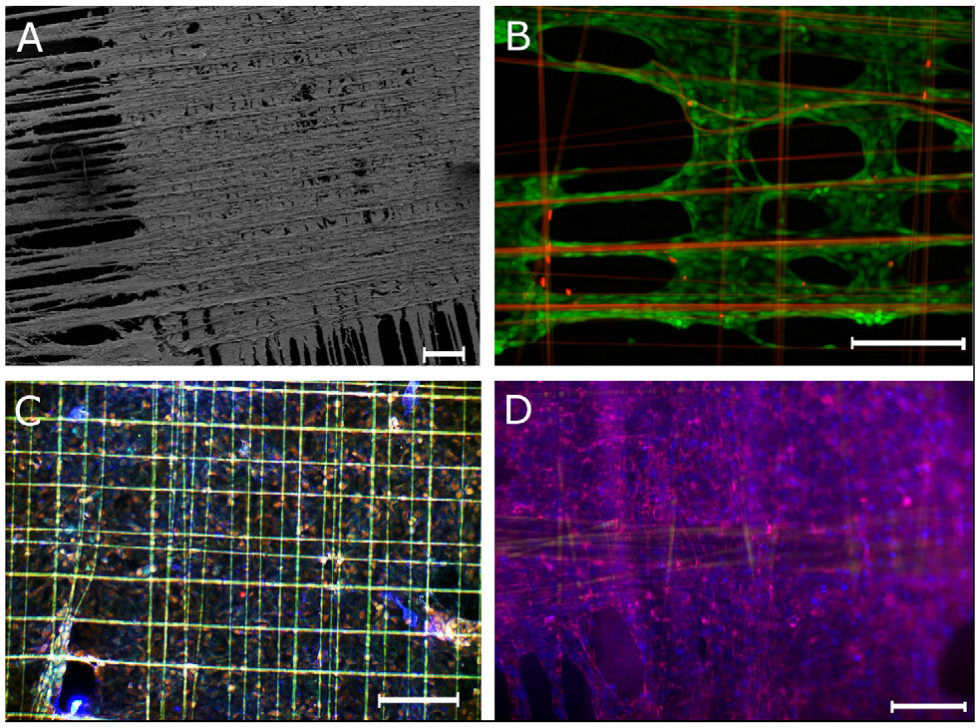
Cell attachment to spider silk fibres on day 3. A: SEM of weaving frame with silk, fibroblasts and extracellular matrix, note the dense central regions; magnitude ×39, scale bar represents 100 µm. B: Live/Dead staining of fibroblasts on silk, assembled in crosslink pattern, viable cells are stained green, dead cells (and spider silk via autofluorescence) red; magnitude ×100, scale bar represents 50 µm. C: Immunofluorescence of spider silk, fibroblasts, and extracellular matrix, Ethidium bromide for staining of the cell nuclei in *orange*, collagen I antibody in *blue*, autofluorescence of spider silk *green*; magnitude ×40, scale bar represents 200 µm. D: Immunofluorescence of spider silk, fibroblasts, and extracellular matrix, DAPI staining of the cell nuclei in *blue*, fibronectin antibody in *pink*, spider silk not visible due to use of secondary antibodies beyond autofluorescence spectrum; magnitude ×40, scale bar represents 200 µm.

The next step was to analyze cytocompatibility of spider silk woven on steel frames by staining with LIVE/DEAD assay after 3 days. Dead cells visibly by red fluorescent nuclei were rarely observed, while the majority of the cells were vital (green fluorescence). The spider silk fibres were ensheaved by spread fibroblasts, forming cell bundles along the spider silk (Fig. 4a).

During further incubation, cells aligned along the silk fibres in a cross pattern and grew mainly in the corners of the crosses. However, with prolongated incubation time the cells were increasingly detected to stretch across the squares.

In long-term-cultivation for 10 and 20 days, a dense green fluorescent layer of green fluorescence and thus of of viable cells was visible (Figure S1).

### Extracellular matrix production occurred

ECM production as reference for metabolic activity after 3 days was shown by immunofluorescence staining with an antibody directed against collagen I (Fig. 4b). Cells and ECM formed laminar, two-dimensional layers, coating the spider silk fibres in a membrane-like manner out of living cells and matrix proteins.

### Adhesion/Proliferation assay displayed both, but lesser than in controls

To determine if changes in adhesion and proliferation occurred between days 1 until 5, weaving frames with spider silk were treated with trypsin to digest surface amino acid residues. As controls, cover slips were coated with collagen and fibronectin.

The variances between the specimens were very high, particularly on the weaving frames (Table 1). Here, cells grew densely on the central parts, in which spider silk fibres were aligned in a crosslink-pattern, while fewer cells appeared on the linear fibres on the peripheral areas of the weaving frame.

**Table 1:** Results of the adhesion/proliferation assay, displayed in cells in fields of vision +/− standard deviation, standard mean error in brackets.

	SpTry (cells/FOV)	Sp (cells/FOV)	Coll (cells/FOV)	Fibro (cells/FOV)
Day 1	57.15+/−41.07 (6.58)	47.83+/−31.46 (5.04)	142.95+/−100.53 (16.10)	76.33+/−90.19 (14.44)
Day 2	82.13+/−52.30 (8.38)	197.80+/−96.36 (15.43)	250.35+/−221.51 (35.47)	569.10+/−646.73 (103.56)
Day 3	164.25+/−135.44 (21.69)	429.38+/−418.42 (67.00)	470.00+/−355.40 (56.91)	503.63+/−455.43 (73.11)
Day 5	970.33+/−596.66 (95.54)	926.23+/−934.03 (149.56)	2485.88+/−761.52 (121.94)	2414.65+/−540.32 (86.52)

FOV = Fields of vision; SpTry = weaving frame with trypsin-digested spider silk; Sp = weaving frame with native spider silk; Coll = weaving frame with native spider silk; Fibro = cover slip with Fibronectin coating.

**Figure 5:**
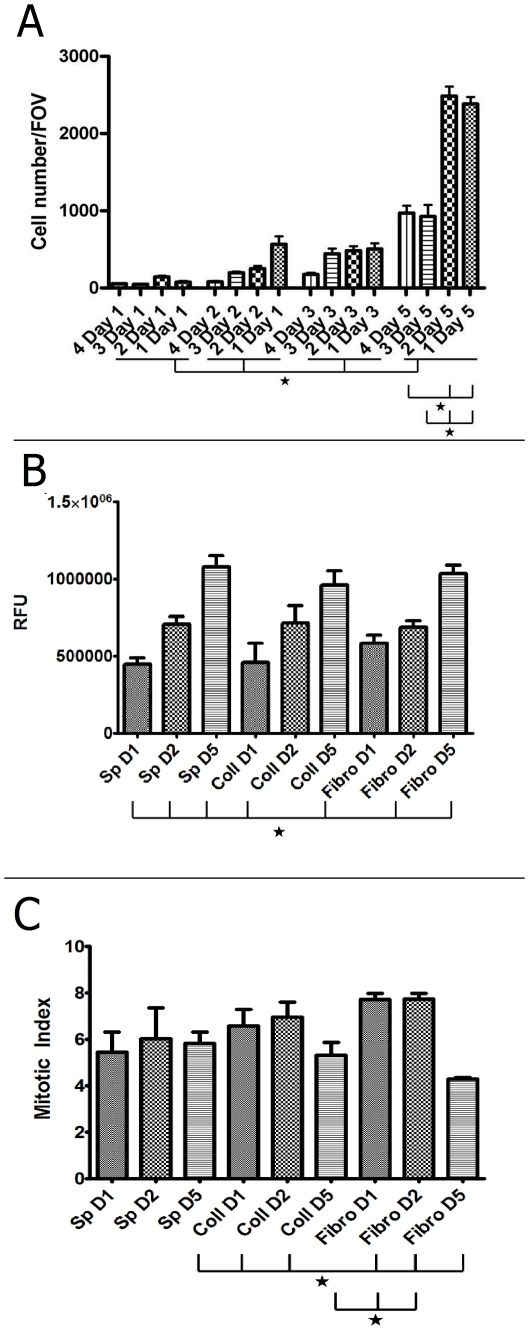
Quantification of time-dependent adhesion/proliferation during incubation. A: Cell count of the proliferation assay, numbers of visible DAPI-stained cell nuclei were counted in fields of vision (FOV) with n = 10 for each specimen, with each specimen treated in quadruplicate. 1 = Collagen-coated cover slip, 2 = Fibronectin-coated cover slip, 3 = weaving frame with native spider silk, 4 = weaving frame with trypsin-treated spider silk. Error bars indicate standard error means, asterisks mark significance level p<0.05. B: Measurement of relative fluorescence of Tubulin in cells either on spider silk weaving frames, collagen-coated controls or fibronectin-coated controls, depicted as relative fluorescent units (RFU). Error bars indicate standard error means, asterisks mark significance level p<0.05. C: Mitotic index of proliferating cells either on spider silk weaving frames, collagen-coated controls or fibronectin-coated controls, calculated by dividing RFU values of Tubulin-positive cells by RFU values of Phospho-Histone H3-positive cells (see text). Error bars indicate standard error means, asterisks mark significance level p<0.05.

Cells were adherent to all of the specimens after 1 day displaying a slightly higher adhesion rate to the fibronectin- or collagen-coated cover slips against the spider silk samples and a higher rate to trypsin-treated spider silk against native spider silk (Fig. 5 a). However, all differences were not significant (p>0.05). These findings were confirmed by staining the cellular tubulin content by specific antibody followed by fluorescent secondary antibody detection as a sensitive measurement for cell growth and proliferation. Cells grown on spider silk displayed slightly though not significantly lower values compared to collagen- and fibronectin-controls (p>0.05, Fig. 5 b).

On day 2, there was a high increase in the controls as well as in the native spider silk. This trend carried on until day three, where again just a slight decrease was visible in collagen-coated cover slips (Fig. 5 a). Cell numbers for native spider silk and fibronectin-coated silk did increased much more, but, again, all differences were not significant (p>0.05). Again, these findings could be confirmed by measuring tubulin content, however, differences between day 1 and 2 were only significant for spider silk (p<0.05, Fig. 5 b).

On day 5, all specimens showed a high increase in cell number, and this time, differences between native spider silk and fibronectin- and collagen-coated cover slips as well as between trypsin-digested spider silk and fibronectin- and collagen-coated cover slips were significant (p<0.01 for both, Table 1, Fig. 5 a). Here, significances were differences between day 1 and day 5 between all samples and between day 2 and 5 for spider silk and fibronectin sample (p<0.05, Fig. 5 b).

**Figure 6:**
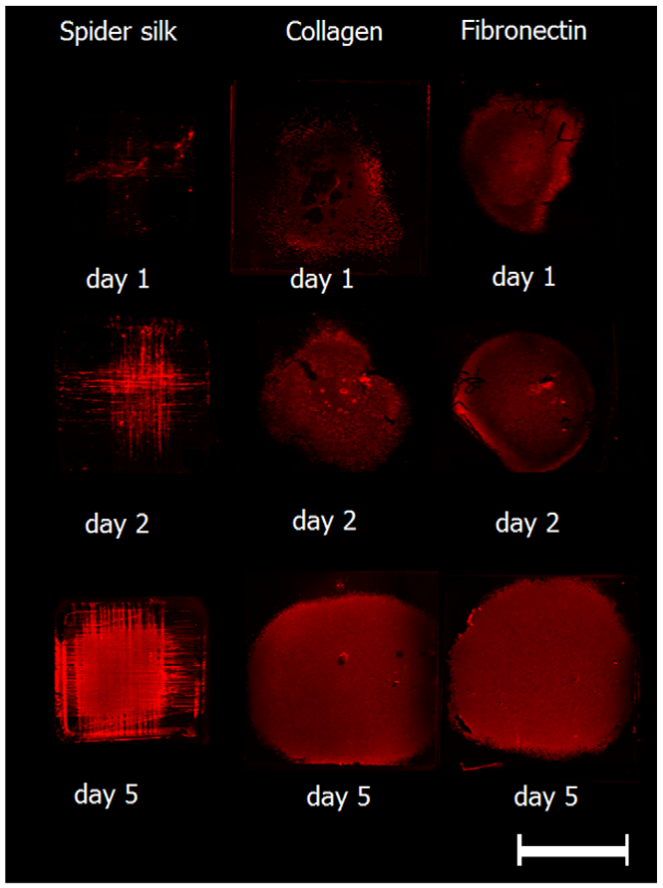
Representative samples of time-dependent relative fluorescence. Immunofluorescence scan of scaffolds seeded with cells on days 1, 2, and 5, stained with Tubulin antibody, columns are spider silk, Collagen control and Fibronectin control; scale bar represents 10 mm.

In Figure 6, a representative set of specimen was scanned to show the fluorescence distribution representing antibody-stained tubulin which shows an overall increase of fluorescence over the incubation period (Fig. 6). The increase was strongest in spider silk samples which initially displayed a small area seeded with cells. On day 5, however, spider silk weaving frames were densely seeded with cells. Mitotic index was constant for controls and spider silk samples (Fig. 5 c). However, these findings were not significant except for the day 5 groups either collagen- or fibronectin controls (p<0.05).

## Discussion

### Summary of results

The weaving frames manufactured in this study together with the network of spider silk woven on them were suitable to achieve a two-dimensional scaffold for the purpose of investigating interactions between spider silk and cells. As they offered regions where fibres crossed rectangular, certain complexity yet simple enough to visualize interactions could be reached.

Our results demonstrate that the observed fibroblasts remained viable, adhering to the spider silk fibres, as well as revealing migratory behaviour. Bundles of cells aligned along the fibres, indicating that contacts were not random. By analysis of cell morphology (cells were spindle-shaped, sticking to the fibres), attachment (fibres were enveloped by cells and ECM), and actin filament bundles (which were orientated along the fibres), we could further substantiate these findings. ECM-production was observed, i.e. cells were embedded in a layer of collagen I (Fig. 4b), a sign for metabolically active cells. In particular, proliferating cultures of NIH/3T3 fibroblasts have been previously described to synthesize amounts of collagen [30].

Analyses was rendered difficult by the fact that spider silk turned out to be autofluorescent in wavelengths between around 400 nm to 630 nm, apparently according to its phosphorus content described by Michal et al. [31], but we were able to avoid too intensive an overlay by using secondary antibodies bordering ultraviolet or infrared spectrum, respectively.

### Adhesion and Proliferation

Adhesion and proliferation could be proven by cell nuclei count over a time period of five days, although proliferation was lower than in the controls (Fig. 5 a). A possible explanation of this may be that the contact area in the controls, the complete cover slip, was a multiple of the surface area offered by the spider silk fibres.

Digesting the spider silk fibres with trypsin resulted in slightly lower cell numbers on days 2 and 3, while on day 5, cell numbers were even higher than on the untreated silk, indicating higher initial adhesion rates in the untreated group followed by higher proliferation rates in the treated groups.

These findings could be supported quantitatively and qualitatively by determination of MI and tubulin content by relative fluorescence measurements. Tubulin was used as ubiquitous cell protein, serving as marker for relative cell content on scaffolds. Initial adhesion on spider silk was slightly less than in controls, probably due to smaller contact area (Fig. 5 b).

Nevertheless, cell on spider silk showed a significant growth on spider silk while growth for control groups was not signifant (p>0.05). Mitotic index was significantly less on day 5 in control groups, probably because of contact inhibition (p<0.05).

### Spider silk surface for adhesion

In our investigation we found that spider silk surface treated for cell culture is very smooth without any visible submicroscopical structures. In recent studies it was shown that spider silk is composed of five layers [16]. The shell is made of a 10–20 nm thick lipid enveloping a 40–100 nm thick glycoprotein layer and an innermost 50–100 nm thick skin layer. The latter consists partly of the main protein, Major Ampullate Spidroin 1 (MaSP1) or Major Ampullate Spidroin 2 (MaSP2), but shows also glycoprotein properties. The two core layers, contributing the major amount of a silk fibre, are composed mainly of MaSP1 and MaSP2.

Whereas the shell can be washed off quite easily with water or detergents, the skin layer and the core layers can be dissolved solely with harsh solvents such as 9 M LiBr, propionic acid/HCL [31], and concentrated formic acid [32]. This procedure destroys the remarkable surface structure by removing the skin layer [16]. As our samples are treated with mild salt solutions used in cell culture we assume that after sterilizing and careful washing, the skin layer (the third layer) remained on top.

### Adhesion mechanisms

The majority of integrins, the proteins mainly responsible for the adhesion of cells to ECM, recognize preferentially aspartatic acid- or glutamic acid-based sequences as ligands (e. g. most common, RGD-residues in fibronectin as well as rarer LDV-, RTD- and KQAGD-residues, but also YRGRD alone as artificial “minimal-peptide”) [28], [29], [33]–[36].

Thus, higher adhesion rates in the Sp group vs. the SpTry group are supposedly due to integrin-mediated adhesion to RGD-residues, which may be mostly cut off enzymatically by trypsin as a protease.

These findings further confirm studies, in which an enhancement of cell attachment could be obtained by using appropriate protein binding sites from biomaterials, i.e. small fragment duplications [37] or RGD-modifications [36]. This has also been demonstrated for spider silk protein [19].

Interestingly, Gellnyck et al. [24] noted an increase in biocompatibility if they treated the silk with trypsin, e.g. less giant cells and fibrosis after 7 weeks, indicating that a change in the immunogenic residues of the spider silk occurred. Possibly, as the adhesion properties of the silk are altered, the ability of immune cells to trigger an inflammatory response decreased as well.

Concerning the increase of proliferation, both by increase of cell numbers as well as ECM production, a magnification of the area serving as adhesive surface was achieved.

We cannot imply that the cells still grow in monolayers after prolonged incubation times which might account for irregular growth rates.

Proliferation rates increased stronger than linearly, possibly until a certain saturation is reached. The maximum number of cells seemed to depend mainly on the individual specimen and thus the amount of spider silk reeled onto the weaving frame.

### Silkworm silk scaffolds

There are a number of publications detailing silkworm silk as matrix material for different tissue engineering purposes. Especially in the most recent articles, impressive results were obtained foremost by the group around D.L. Kaplan [38], [39], but also by Mandal & Kundu [40].

Among many other applications, it has also been used as a material for biomedicine, e.g. as a suture material.

However, virgin silkworm silk is known to be immunogenic, causing inflammatory reactions *in vitro* and *in vivo*, including asthma attacks [41]–[44]. According to common belief, sericin is responsible for this reaction, a glycoprotein enveloping fibroin, the actual silk protein. If silkworm fibres are “degummed”, i.e. separated from this sericin coating, immunologic reaction is considerably less [43].

Nevertheless, this kind of fibroin production is complicated, as it needs to be dissolved yielding an aqueous-derived protein that in turn has to be moulded by salt-leeching, freeze-drying, and, finally, air-drying [38], [39], [45], [46].

With the method developed in this study, two-dimensional scaffolds can be designed with far less technical complexity. By using a steel weaving frame, the disadvantages of supercontraction could be avoided, which were considered as obstacle for biomedical applications [27]. The system is stabile and can be adapted to different applications.

### Biomedical use of spider silk

A coating of cell culture surfaces with a recombinant spider silk protein resulted in an increase of proliferation rates, supporting adhesion of cells although the spider silk protein was coupled to elastin [25]. Yet our study is the first that investigated a two-dimensional model using native spider silk fibres. While a recent study found a mild decrease in proliferation rates of endothelial cells that were exposed to spider silk [26], it has to be mentioned that the spider silk utilized was yielded from *Nephila edulis*. Additionally, in that study spider silk was used neither as coating nor as scaffold.

Whereas spider silk fibres, because of their complex composition, could as yet not be produced *de novo*, spider silk proteins, i.e. MaSP1 and MaSP2 have been obtained from bacteria [47], insect cells [48], plants like tobacco or potato-plants [49], and goats [50]. These proteins can be extracted dissolved in an aqueous solution. Spinning these proteins into high-performance fibres comparable to those produced by the spiders' glands still remains a challenge due to the complex dehydration process performed in the glandular duct [17]. While great advancements may be expected with this problem in the future [18], there is a paucity of data dealing with the concrete use of native spider silk for tissue engineering purposes. Therefore, our study has a pilot role describing the use of native spider silk fibres as a biomaterial, in particular as scaffold weaved on a weaving frame, potentially rivalling other biomaterials currently in use.

### Conclusion

Among many materials used for tissue engineering and investigation of cell interactions, we believe our study describes a biomaterial that can easily be harvested and designed to use as a scaffold. The woven cross-pattern displayed a simple alignment to investigate cell interactions, whereas more complex structures may conceal those processes. Furthermore, owing to the cytocompatibility and the mechanical strength of the dragline silk, it can be utilized as tissue engineering matrix that can replace the function of the desired tissue after implantation. With the method presented here, basic science studies with native spider silk fibres are easier to perform and visualize than common attempts. For the first time, adhesion as well as proliferation on spider silk could directly be visualized and determined in this study.

As some of the mechanisms of cell attachment to spider silk fibres remain poorly understood, the remarkable surface properties of spider dragline silk merit future investigation. Furthermore, tissue engineering of more complex structures should be carried out, as spider silk as a biomaterial can rival most artificial matrices, as well as silkworm silk.

## Materials and Methods

According to the German Animal Welfare Law as well as the Directives of the European Union, spiders as invertebrates do not require any approval.

### Preparation of culture dishes and layout of the assays

6-Well-Plates (TPP, Trasadingen, Switzerland) were rinsed with 0.2% (w/v) Pluronic F-127 (Sigma-Aldrich, Bomem, Belgium) in phosphate buffered saline (PBS; PAA, Pasching, Austria) w/o Ca^2+^/Mg^2+^ for 1 hour to be non-adhesive for cells [51]. For testing purposes, we also treated the spider silk in this manner, but no influence on adhesion properties could be observed (data not shown).

In all assays, experiments were carried out with specimens in duplicate, except adhesion/proliferation assays, which were carried out in quadruplicates.

### Rearing of the spiders

The spiders of the species *Nephila clavipes* were kept in rooms with up to 15 animals per room to avoid cannibalism. Webs were sprayed with tap water every day. Additionally, vaporizers were used to moisten the air. Spiders were fed with crickets (*Acheta domesticus*) three times per week. Only adult female spiders were used for silking and fed an extra time after silking. For silk harvest we used a method described earlier with little modifications [52]. Briefly, the spiders were atraumatically fixed and immobilized on styrophor cubes with gauze and needles, and the major ampullate gland was stimulated by pulling the dragline out of the anterior spinneret mechanically. Spiders were not harmed during the harvesting process and no anaesthesia was used to avoid pH changes induced by carbon dioxide (CO_2_) anaesthesia [53].

### Manufacture of weaving frames

With stainless steel wire in a thickness of 0.7 mm purchased from a dental technique manufacturer, we bent small weaving frames in sizes ranging from 5 to 20 mm (REF 527-070-00, Dentaurum, Ispringen, Germany). The frames were sterilizable and inert in cell culture conditions as an imporatant prerequisite for use in cytocompatibility testing. Using motorized drum system with a specially designed device, the frames could be provided with a network of fibres (Fig. 1a) aligned in an even pattern with spaces between 50 and 250 µm (Fig. 1b). Woven frames were rinsed with 70% Ethanol and autoclaved by steam sterilizing at 121°C, 2 bar, and 100% water saturation for 15 minutes after weaving.

### Controls

For controls, cover slips were either rinsed with 70% Ethanol, autoclaved and coated with 0.15 mg/ml fibronectin solution in PBS (Biochrom AG, Berlin, Germany) for 30 minutes at room temperature (RT) and washed with PBS, or were covered with 1 mg/ml collagen A (Biochrom AG) solution for 30 minutes at 37°C and washed with PBS, respectively.

### Cell culture and seeding

NIH/3T3 cells were cultured in Dulbecco's modified Eagle's medium (DMEM) High Glucose Cell culture medium (PAA) supplemented with 10% fetal calf serum (FCS)(Biochrom AG), 1% Sodium-pyruvate (PAA) and 1% Gentamycin solution (10.000 µg/ml; Biochrome AG, Berlin, Germany). Weaving frames were placed on the bottom of the Pluronic-coated culture dishes and seeded with 5×10^5^ cells/ml for SEM and 5×10^3^ cells/ml for other investigations. Cells were dripped carefully on the specimens and then incubated at 37°C at 95% humidity/5% CO_2_. Cells were observed daily by light microscopy and the cell culture medium was changed on days 3 and 5.

### Viability staining and SEM

For viability staining, LIVE/DEAD cell viability assay® (Invitrogen, Carlsbad, CA, USA) was used on days 3, 10, and 20, following manufacturer's guidelines. After incubation, cells were viewed with an inversed fluorescence microscope and photographed with AxioVision® software (both from Carl Zeiss, Jena, Germany).

For SEM, specimen were fixed in Sodium-Cacodylate buffer, pH 7.3 (Merck, Darmstadt, Germany) containing 2.5% Glutaraldehyde (Polysciences, Warrington, P.A., U.S.A) for 24 hours, dehydrated by insertion in increasing acetone dilutions and dried with a CPD030 (Bal-Tec, Balzers, Liechtenstein), followed by gold sputtering with a SEM Coating System (Polaron, East Grinstead, United Kingdom). Specimens were put in a vacuum, viewed in a SEM500 (Philips, Hamburg, Germany). Photographs of the views were taken with a method and software developed by Gebert & Preiss [54].

### General treatment for Immunofluorescence

Incubation of the cells was stopped after 24 h, cell growth was examined with light microscopy and cells were fixed with 4% paraformaldehyde (PFA, Sigma-Aldrich) for 20 minutes at room temperature. The cell membrane was perforated with 0.1% Triton X-100 (Sigma-Aldrich) in PBS for 4 minutes and blocked with 2% FCS in PBS for 30 minutes at RT. Primary and secondary antibodies were applied for 60 minutes at 37°C, followed by extensive washing. Specimens were covered in Vectashield® (Vector laboratories, Burlingame, CA, USA), viewed and photographed using AxioVision®.

### Characterization of the adhesion

To determine cytoskeleton-assembly, we stained α-Actin filaments with Phalloidin-Alexa 488 (Invitrogen) conjugate at a dilution of 1∶500 and used 4′,6′-di-amidino-2-phenyl-indol (DAPI, Vector laboratories) to visualize the nuclei.

### Immunofluorescence detection of the ECM

In order to determine if fibroblasts produce extracellular matrix (ECM), an immunostainining for endogenous collagen I was performed which served as a representative for cell metabolism and secretion.

Polyclonal collagen I antibody (derived from rabbit; Abcam, Cambridge, MA, USA) was used as primary antibody at a concentration of 1∶40, secondary antibody was Alexa 350 goat anti-rabbit (Invitrogen) at a concentration of 1∶500.

Ethidium Bromide (Invitrogen) at a dilution of 1∶50,000 was used for labelling the cell nuclei.

Because spider silk is prone to strong auto-fluorescence signals, Alexa 488 goat anti-rat (Invitrogen) was added to the samples to mark the spider silk fibres by unspecific attachment. We ruled out unspecific binding to the cells by previous blocking, so spider silk could be distinguished by fluorescence emission at the appropriate wavelength.

### Adhesion and proliferation assay

Spider silk-woven frames were treated with trypsin (bovine Pancreas, 12,400 U/mg, Sigma-Aldrich, Bornem, Belgium) at a concentration of 1 mg/ml for 4 h at 37°C. Treated frames (SpTry), control frames with untreated spider silk (Sp) and fibronectin- (Fibro) as well as collagen-coated (Coll) controls were incubated for 1, 2, 3, and 5 days, respectively. After cell membrane perforation, we mounted the specimens with Vectashield® including DAPI for staining of cell nuclei. The nuclei were counted in 10 randomly chosen fields of vision (FOV) for each specimen at a magnification of ×40 using an inverse fluorescence microscope and visualizing software (Carl Zeiss).

As second proliferation assay, an immunostaining with primary antibody against Tubulin (derived from rat, Abcam) at a concentration of 1∶400 and secondary antibody Alexa 680 goat anti-rat (1∶4,000, Invitrogen) was performed after 1, 2, and 5 days, RFU were measured with LiCor Odyssey Infrared scanner (LiCor Germany GmbH, Bad Homburg, Germany). Each proliferation assay was carried out triplicate in two different experiments.

Additionally, in the same specimens, mitotic cells were stained with Phospho-Histone H3 antibody (1∶200, derived from rabbit, Abcam) and LiCor 800 donkey anti-rabbit infrared antibody (1∶4,000, LiCor) and mitotic index (MI) was calculated by dividing RFU for Tubulin-positive cells by RFU of Phospho-Histone H3-positive cells for each day:
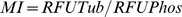


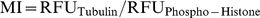
The median, standard deviation (SD), and the standard mean error (SM) was calculated with Microsoft Excel, testing for statistical significance was done with student's t-test and the results were analysed for variance with Analysis of Variance (ANOVA) followed by Bonferroni's post-hoc correcture.

## Supporting Information

Figure S1 Long-term viability of fibroblasts on spider silk. A, B: Representative samples Live/Dead staining of fibroblasts on silk, viable cells are stained green, dead cells (and spider silk via autofluorescence) red; magnitude ×40, scale bar represents 200 µm.
(6.65 MB TIF)
