# Supplementary figures and images for: Interactions between Spider Silk and Cells – NIH/3T3 Fibroblasts Seeded on Miniature Weaving Frames
Source: PLoS One. 2010 Aug 9;5(8):e12032. doi: 10.1371/journal.pone.0012032 (PMC2918503; doi:10.1371/journal.pone.0012032)

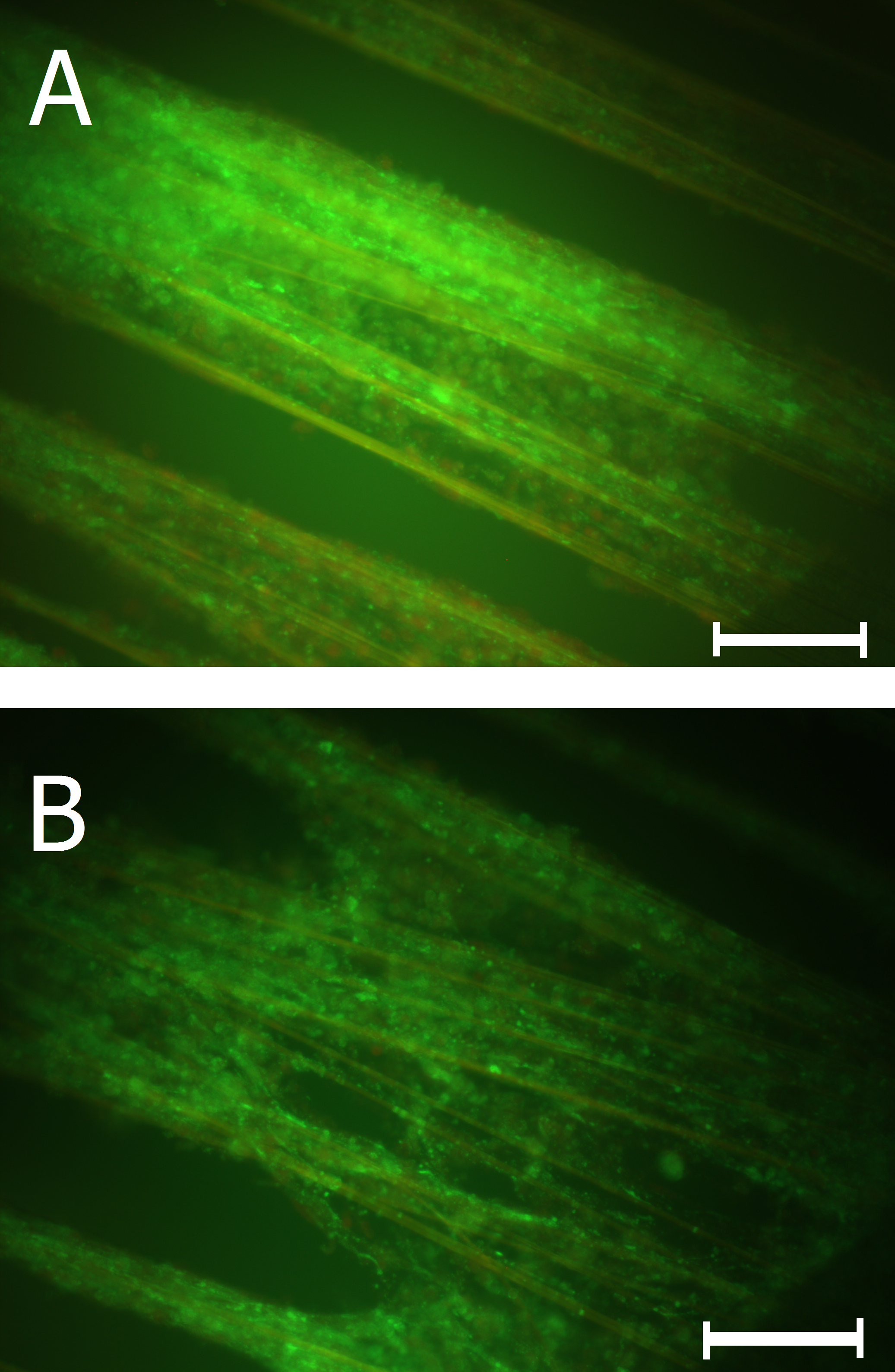

Supplement: Figure S1 — Long-term viability of fibroblasts on spider silk. A, B: Representative samples Live/Dead staining of fibroblasts on silk, viable cells are stained green, dead cells (and spider silk via autofluorescence) red; magnitude ×40, scale bar represents 200 µm. (6.65 MB TIF) [file pone.0012032.s001.tif]
